# Sleep Quality and Fatigue among Breast Cancer Patients Undergoing Chemotherapy

**Published:** 2019-10-01

**Authors:** Mahrokh Imanian, Mahsa Imanian, Mahdi Karimyar

**Affiliations:** 1 Medical Surgical Nursing, Jahrom University of Medical Sciences, Jahrom, Iran; 2 Student Research Committee, Shiraz University of Medical Sciences, Shiraz, Iran

**Keywords:** Sleep, Fatigue* Breast, Cancer, Chemotherapy

## Abstract

**Background:** Breast cancer survivors make up a growing population facing treatment that poses long – standing adverse effects including chemotherapy- related sleep disorders and fatigue. There is limited knowledge of patients' lived experiences of chemotherapy- induced sleep disorders and fatigue. The aim of this study was to explore sleep quality and fatigue among breast cancer patients undergoing chemotherapy.

**Materials and Methods:** One hundred fifteen patients were included in this census-based cross-sectional study. Data were collected through the Pittsburgh Sleep Quality Index and Brief Fatigue Inventory four days after the chemotherapy session**. **Statistical analysis was carried out using SPSS software version 13 and P<0.05 was considered statistically significant in all tests.

**Results:** The mean hours of sleep were 5.6±1.83 in the range of 2 to 10 hours. The mean score of fatigue of participants was 5.59±1.67. Based on the cutting point, 57.4%, 20.9%, and 21.7% of participants had a moderate (4-6.9), mild (0.1-3.9), and severe (7-9.9) level of fatigue, respectively. The mean score of sleep quality among the participants was equal to 14.06±3.06, with a maximum and minimum of 7 and 21. The results of Spearman correlation coefficient showed that there is a significant relationship between fatigue and quality of sleep )0.210).

**Conclusion:** Although the study findings revealed that patients with breast cancer undergoing chemotherapy experience different degrees of sleep disorders and fatigue, there is a need for more detailed studies to improve the quality of sleep and reduce fatigue in these patients due to the little attention paid to this issue in the medical field.

## Introduction

 Breast cancer is the most prevalent cancer among women worldwide^1^. It is reported that 25% of diagnosed cancers in women are known to be breast cancer and 15% of overall deaths are caused by it^   2 ^. The incidence of breast cancer annually increases by 5% in low- and middle-income countries, as this disease is considered an emerging public health emergency ^3^. 

In Iran, based on National Center for Cancer Registration report, breast cancer constitutes 23% of all the cancers diagnosed in women^   4 ^. In comparison with developed countries, breast cancer occurs a decade earlier in Iranian women^   5 ^. Cancer diagnosis and treatment are associated with a high level of mental and psychological problems. In fact, in the Middle East, cancer diagnosis has remained a taboo for patients, which predisposes them to mental disorders^6^. Fatigue and sleep disorders are said to be the most common and annoying symptoms of cancer patients. About 70-80% of newly-diagnosed patients or those who have recently undergone treatment suffer from fatigue and 30-75% of them complain of sleep disorders^7^. In addition, the prevalence of fatigue among patients under radiotherapy has been reported to be 60-93%^8^. Studies have shown that sleep disorders sometimes lead to fatigue and depression before and during treatment. Moreover, sleep quality impairment is associated with a decrease in quality of life, decreased function, more pain, decreased energy, and more mental and health problems^9^. Researchers argue that sleep disorders and fatigue caused by cancer are associated with many factors including anemia, fever, pain, weight loss, infection, and depression^7^. On the other hand, activation of inflammatory responses in the body and disruption of the circadian cortisol rhythm negatively affect the sleep-wake cycle^9^. Researchers believe that fatigue and sleep disorder caused by cancer are an important part of the category of Sickness Behaviors^7^. There are pieces of evidence that sleep disorders worsen after the beginning of chemotherapy^9^. In addition, cancer treatment is associated with pain, fatigue, and changes in the body's biological rhythm^7^. If fatigue is not treated effectively and timely in cancer patients, it can lead to anxiety, depression, and decreased quality of life^10^. Studies show that cancer-induced fatigue persists for up to 10 years or more after initial diagnosis leads to impairment in work, sleep, and social relationships and reduced quality of life^11^. In this regard, two cross-sectional studies in Bahrain and Jordan showed that fatigue and sleep disorders are the most annoying symptoms reported by women with breast cancer under treatment^12^^,^^13^. Another cross-sectional study conducted in Jordan indicated that fatigue is the most annoying symptom among women with cancer under chemotherapy^14^. There is little information in Iran about the sleep-wake cycle fatigue in patients with breast cancer, especially in the early stages of chemotherapy treatment. Hence, considering the importance of the issue and the need for planning nursing care to treat these two annoying side effects, the present research aims to study the quality of sleep and fatigue among patients with breast cancer undergoing chemotherapy.

## MATERIALS AND METHODS

 A cross-sectional study was performed between April to June 2013 at the Hematology clinics of Shafa and Golestan Hospital affiliated with Ahwaz Jundishapur University of Medical Sciences. The study population consisted of all breast cancer patients over the age of 25-65 years old referred to these centers. Sampling was performed using a census method. The inclusion criteria were patients with stage 0–II breast cancer in the 25-65 age range receiving 1-5 cycles of chemotherapy, able to complete the questionnaires and no history of psychiatric disorders or amnesia. The diagnosis had been made within 1-5 months prior to entry in the study and all patients received equal regimen.

Patients who did not wish to participate the study were excluded. Finally, 115 patients were entered the study.

**The study instruments**

Data were collected through three questionnaires. The first of the above-mentioned questionnaires was about demographic data of age, education level, marital status, job and medical history. 

The Pittsburg sleep quality Index (PSQI) was used to evaluate the quality and the disturbances of sleep in a recent one-month period. The PSQI contains 19 questions and its global score varies from zero to 21. Higher values indicate lower of sleep quality. Score of five constitutes the cut-off point allowing one to distinguish between subjects with poor and good sleep quality^   15 ^.

The third questionnaire (scale) was Brief Fatigue Inventory (BFI). The BFI consists of nine items about unusual fatigue felt during the past week, including three items about the severity, “usual” and “worst” level of fatigue during last 24 h and six items that measure the amount of fatigue interference in life aspects during the past 24 h. The interference items are measured on a 0–10 scale, with zero being “does not interfere and 10 being “completely interferes”. In previous studies, the BFI demonstrated a strong internal consistency coefficient of 0.96^   16 ^. 

Four days after the chemotherapy session, the subjects were given the questionnaires.

**Data collection**

The researcher presented in the Hematology clinics, assessed potential participants for eligibility, briefed the eligible subjects on the study purposes, and conducted data collection. Data were collected through individual interviews.

**Ethical Consideration**

This study was approved by Ethics Committee of Ahwaz University of Medical Sciences (Registration number ajums.REC.1392, 36). All participants signed a consent paper before their participation in the study and were assured of the anonymity and confidentiality of their personal data. 

**Data Analysis**

Statistical analysis was carried out using SPSS software version 13(SPSS Inc., Chicago, IL, USA). The Kolmogorov-smirnov test was used to examine the normal distribution of variables. Descriptive statistics (i.e., frequencies, percentage, mean and standard deviation) were calculated. Independent samples t-test was used to compare the mean scores of sleep quality and fatigue. Pearson and spearman correlation coefficients were calculated to examine the relationship between sleep quality and fatigue. P<0.05 was considered significant in all tests.

## Results

 The study sample consisted of 115 patients with breast cancer. The mean age of participants was 47.03±10.24 years with an age range of 25 to 69. The mean number of cycles of chemotherapy was equal to 3.83±2.27, with a minimum and maximum of 0 and 5. In addition, the mean hours of sleep were 5.6±1.83, in the range of 2 to 10 hours. According to the obtained data, 27.8% of participants were taking painkillers. 

Demographic variables of patients with breast cancer (Table 1) show that 89.6% of participants were unemployed and 77.7% of them had a total type of surgery. The results also indicated that 90.4% of patients had a high school diploma or a lower educational degree. In terms of marital status, 52.2%, 38.6%, and 9.6% of participants were married, single, and widow, respectively. In addition, 35.7%, 46.1%, and 1.7% of them had a familial history of other diseases, cancer, and psychiatric diseases, respectively. 

The mean score of fatigue of participants was 5.59±1.67, in the range of 1.91 to 8.73. Based on the cutting point, 57.4%, 20.9%, and 21.7% of participants had a moderate (4-6.9), mild (0.1-3.9), and severe (7-9.9) level of fatigue, respectively. 

The mean score of sleep quality among the participants was equal to 14.06±3.06, with a maximum and minimum of 7 and 21. Higher scores indicate lower quality of sleep. 

The results of Spearman correlation coefficient (Table 2) showed that there is a significant relationship between fatigue and quality of sleep )0.210). This acknowledges that the sleep quality score increases in breast cancer patients with the increase in fatigue score, and increased sleep quality score indicates lower quality of sleep.

The results of Spearman correlation coefficient (Table 2) demonstrated that fatigue has a significant relationship with mental quality of sleep (r=0.216(, sleep duration (r=-0.24), sleep efficiency )r=-0.202), sleep disorders(r=0.353), taking sleeping pills(r=0.241) and daily functional disorders(r=0.374). This indicates that mental quality of sleep, sleep disorders, taking sleeping pills, and daily functional disorders increase in breast cancer patients with the increase in the fatigue score. By contrast, sleep duration and sleep efficiency reduce as the fatigue score increases.

The results of statistical analysis showed that demographic variables of breast cancer patients have no significant relationship with fatigue and sleep quality (p >0.05).

**Table 1:** Demographic variables of patients with breast cancer

**variable**		**N**	**Percent**	**variable**		**N**	**Percent**
Type of surgery	Unilateral mastectomy	24	21.4%	History of other disease	Yes	41	35.7%
	Breast-conserving surgery Both of breast mastectomy	87 1	77.7% 0.9%		No	74	64.3%
				History of cancer in family	Yes	53	46.1%
Educational level	High school	51	44.3%		No	62	53.9%
	Secondary	17	14.8%	Employment status	Unemployed	103	89.6%
	Diploma	36	31.3%		Employed	8	7.0%
	Associate’s degree	2	1.7%		Retired	4	3.5%
	Bachelor’s degree	7	6.1%	History of mental disease	Yes	2	1.7%
	Master science	2	1.7%		No	113	98.3%
Marital status	Married	60	52.2%	Taking opioid or sleeping pills	Yes	0	0.0%
	Single	44	38.3%		No	115	100.0%
	Divorced or widowed	11	9.6%				

**Table 2:** The relationship between the research variables and sleep quality parameters in patients with breast cancer (Spearman correlation coefficient)

	**Mental quality of ** **sleep**	**Delay in falling ** **asleep**	**Sleep duration**	**Sleep efficiency**	**Sleep ** **disorders**	**Taking sleeping pills**	**Daily functional ** **disorders**
Fatigue	0.216*	0.161	-0.24*	-0.202*	0.353*	0.241*	0.374*

## Discussion

 With an increase in the incidence of cancer in recent years and its impact on physical, psychological, and social dimensions, it is considered a major health problem of the century^1^. The present research aimed to study the quality of sleep and fatigue among patients with breast cancer undergoing chemotherapy. The participants aged 25-69 years. The results showed that the mean score of sleep quality among the patients was equal to 18.03±5.97. Some studies have reported the high level of sleep disorders in women undergoing chemotherapy ^2^^, ^^3^. In a study conducted by Saini, the prevalence of undesirable sleep quality in patients undergoing chemotherapy was reported to be 58.8%^4^. Whereas the onset of insomnia begins after the diagnosis of breast cancer for some patients, some others have reported that cancer has caused or aggravated their sleep problems^5^. In the present study, the quality of sleep decreased in patients with the increase in the sleep quality score, with a mean sleep duration of 2-10 hours. In addition, the results of this study indicated the extremely high prevalence of fatigue in patients. In a comprehensive study conducted on 763 patients with breast cancer, it was shown that 34% and 35% of women suffered from fatigue between 5-10 and 1-5 years after diagnosis and 21% of them experienced fatigue in both evaluations. This suggests that cancer-related fatigue be severe and persistent^6^. In this study, the majority of patients (57.4%) had a moderate level of fatigue and 21.7% and 20.9% of them suffered from severe and mild levels of fatigue, respectively. Some studies have reported that the prevalence of fatigue during the treatment ranges between 25 and 95^7^^, ^^8^. In a study conducted by Mehri on chemical war veterans, the mean score of fatigue was equal to 81.6±15.4 ^9^. The study findings also indicated that there is a significant relationship between fatigue and pain severity and also between fatigue and sleep quality. This acknowledges that pain severity increases and sleep quality reduces with the increase in the fatigue score. The results of Alexander showed that women with breast cancer suffering from fatigue significantly experience higher duration of sleep and more nightly wake compared to breast cancer patients without fatigue^10^. Considering the study results, it seems that the prevalence of fatigue can be reduced and the treatment process can be accelerated by improving sleep quality in patients undergoing chemotherapy.

## CONCLUSION

 Although the study findings revealed that patients with breast cancer undergoing chemotherapy experience different degrees of sleep disorders and fatigue, there is a need for more detailed studies in order to improve the quality of sleep and reduce fatigue in these patients due to the little attention paid to this issue in the medical field. 
